# Research on immunity and ageing comes of age

**DOI:** 10.1186/s12979-019-0148-9

**Published:** 2019-04-29

**Authors:** Nan-ping Weng, Graham Pawelec

**Affiliations:** 1 Laboratory of Molecular Biology and Immunology, National Institute on Aging, National Institutes of Health, Bethesda, USA; 2 Department of Immunology, University of Tübingen, Tübingen, Germany; 3 Health Sciences North Research Institute, Sudbury, ON Canada

**Keywords:** Age, Immunity

## Abstract

Ageing has a profound detrimental impact on almost all living organisms. Immune systems play a particularly important role in protection against external challenges (pathogens) and internal insults (cancer) but their protective capacity commonly wanes with advancing age. With the rapid increase in the numbers of older people around the world, research in the field of immunity and ageing is becoming increasingly important. This realization, together with recent and ongoing technical advances in analytical capabilities, is facilitating rapid progress towards a better understanding of immunity and ageing and the resulting anticipated improved application of this knowledge to medical treatments in the years ahead.

Studies on immunity in older individuals, especially humans, are progressing rapidly as awareness of the importance of immune system ageing for the integrity of the entire organism becomes increasingly apparent. The continuing growth of the population of older people in many countries around the world has further emphasized the importance of immune ageing as a crucial field of study. Recent technological advances allow the exploration of new areas of immunity and age-associated changes at the gene, cell, organismal and societal level, whereas the increasing numbers of older people raises awareness of the requirement for more funding support from governments and private sources.

Over the past decade, we have witnessed a transformation in research on immune system ageing from mostly a descriptive and cellular phenotypic characterization based on in vitro research to more mechanistic and multi-level investigations both in vitro and in vivo. *Immunity and Ageing* will remain at the forefront for publishing advances in all immunity and ageing research with particular emphasis on emerging areas such as, for example 1) Epigenetic changes and their consequences for immune function. By regulating chromatin status, epigenetic mechanisms control gene transcription during immune cell development and differentiation. Aging alters lymphocyte functions that may be rooted in part in the proper establishment and maintenance of the chromatin in immune cells. Elucidating epigenetic regulation and its changes during aging will shed new light on the mechanisms of immune cell aging and open new avenues for clinical interventions. 2) Alteration of metabolic status in immune cells during aging is becoming an area of intense study partly as a result of technical advances in analytical capabilities. It has long been recognized that nutritional and metabolic factors have a profound impact on healthspan and lifespan. However, the mechanisms underlying the nutritional and metabolic impact on immune cell development, differentiation and functions, are only recently beginning to be understood. Much more needs to be learned about the precise changes in mitochondria, metabolites, and their interactions with aging. Such knowledge is essential for understanding the changes of immune functions during ageing and is likely applicable to clinical interventions as well. 3) Inflammation is one of the most widely-recognized aging phenomena in mammals. It reflects not only dysregulation of innate and adaptive immune cells (B and T lymphocytes) but also non-immune cells. Although circulating inflammation-related cytokines have been measured using advanced methodology, the precise causes and consequences of increased systemic levels of inflammation-related cytokines remains to be elucidated. As “inflammaging” has been implicated in many age-related pathologies including neurodegenerative diseases and cardiovascular diseases, understanding the mechanisms involved will lead to better understanding of these pathologies and better clinical diagnosis and potential treatment. These 3 examples, but also many other areas of fruitful research in humans and model organisms will be profoundly important for the future wellbeing of ageing societies. Longitudinal studies in humans, and better animal models for age-associated alterations in more natural “real life” environments are expected to play an increasingly important role in determining bases for rational interventions to appropriately improve immune function.

*Immunity and Ageing* is the only specialist journal focused exclusively on this important area and will continue to publish crucial advances in the field. Now with a new editorial board consisting of leading experts in research on immunity and ageing, the Journal will build on the firm basis developed by the outgoing Founding Editor, Prof. Calogero Caruso, and will be the go-to place for publishing and learning about the most recent advances related to the phenomena and responsible mechanisms underlying the development, differentiation, and ageing of immune cells and functions, and for the most up-to-date reviews of important areas of immunity and aging research. We welcome ideas and proposals for making *Immunity and Ageing* an even better journal for researchers and readers who are interested in all aspects of ageing and immune function in humans and model organisms.

